# Effect of Probiotic Bacteria on the Gut Microbiome of Mice with Lipopolysaccharide-Induced Inflammation

**DOI:** 10.3390/microorganisms12071341

**Published:** 2024-06-30

**Authors:** Mariya Gryaznova, Inna Burakova, Yuliya Smirnova, Polina Morozova, Egor Chirkin, Artem Gureev, Evgeny Mikhaylov, Olga Korneeva, Mikhail Syromyatnikov

**Affiliations:** 1 Laboratory of Metagenomics and Food Biotechnology, Voronezh State University of Engineering Technologies, 394036 Voronezh, Russia; mariya-vg@mail.ru (M.G.); vitkalovai@inbox.ru (I.B.); dyd16@mail.ru (Y.S.); ms.cloud00.00@mail.ru (P.M.); korneeva-olgas@yandex.ru (O.K.); 2 Department of Genetics, Cytology and Bioengineering, Voronezh State University, 394018 Voronezh, Russia; hirkin01222004@yandex.ru (E.C.); gureev@bio.vsu.ru (A.G.); 3 FSBSI All-Russian Veterinary Research Institute of Pathology, Pharmacology and Therapy, 394061 Voronezh, Russia; voronezh81@rambler.ru

**Keywords:** mice, LPS, *Lactobacillus plantarum*, *Weissella confusa*, gut microbiota

## Abstract

The role of lipopolysaccharide (LPS) in the development of diseases is clear, but the specific mechanisms remain poorly understood. This study aimed to investigate the microbiome aberrations in the guts of mice against the background of LPS, as well as the anti-inflammatory effect of probiotic supplementation with *Lactobacillus plantarum* from the gut, a mix of commercial probiotic lactic acid bacteria, and *Weissella confusa* isolated from milk using next-generation sequencing. LPS injections were found to induce inflammatory changes in the intestinal mucosa. These morphological changes were accompanied by a shift in the microbiota. We found no significant changes in the microbiome with probiotic supplementation compared to the LPS group. However, when *Lactobacillus plantarum* and a mix of commercial probiotic lactic acid bacteria were used, the intestinal mucosa was restored. *Weissella confusa* did not contribute to the morphological changes of the intestinal wall or the microbiome. Changes in the microbiome were observed with probiotic supplementation of *Lactobacillus plantarum* and a mix of commercial probiotic lactic acid bacteria compared to the control group. In addition, when *Lactobacillus plantarum* was used, we observed a decrease in the enrichment of the homocysteine and cysteine interconversion pathways with an increase in the L-histidine degradation pathway.

## 1. Introduction

Lipopolysaccharide (LPS or endotoxin) is a thermostable component of the outer part of the cell membrane of most Gram-negative microorganisms [1]. This macromolecule can lead to dysregulation of the immune system, which plays an important role in the development of a wide range of pathologies, including neurodegenerative, metabolic, and cardiovascular diseases [2].

LPS consists of three covalently linked components: lipid A, which after the destruction of the bacterial cell is released into blood and can have a severe toxic effect; a central oligosaccharide, which is also an endotoxin and, when released into blood, causes a toxic effect, but to a lesser extent than lipid A; and O-antigen is the most immunogenic part of LPS, which is easily recognized by the host immune system [3].

By its nature, LPS is not toxic, but its effect is mediated by the activation of the immune system and depends on many factors that alter the host’s susceptibility [4]. Small amounts of LPS are released from the surface of bacteria, while large amounts are released upon bacterial cell lysis or death [5].

LPS is involved in the development of various diseases; for example, its role has been shown in Parkinson’s disease, Alzheimer’s disease [6], diabetes, obesity [7], and atherosclerosis [8], among others.

The search for a therapeutic approach is mainly associated with the neutralization of LPS or the inhibition of LPS-mediated activation of immune cells. However, such experiments did not lead to the expected positive results [3]. The intestinal microbiota is capable of producing various metabolites, regulating the number of pathogenic bacteria, and modulating the immune response, for example, by reducing intestinal permeability to LPS [9]. All of this suggests that taking probiotic bacteria may be able to correct the toxic effect caused by LPS.

Probiotic supplements are known to have the ability to restore gut microbiome composition and impart beneficial functions to host gut microbial communities, which in turn reduces or prevents the development of intestinal inflammation and other intestinal or systemic disease phenotypes [10].

*Lactobacillus plantarum* is a widely used probiotic with specific beneficial immunoregulatory properties, including regulation of the immune response [11]. In general, bacteria of the genus *Lactobacillus* are capable of modulating the immune system and exerting anti-inflammatory effects [12]. Although some studies indicate that the bacterium *Weissella confuse* can cause sepsis and other serious infections in humans and animals [13], it is often considered as a probiotic supplement, as it has high survival rate in gastrointestinal diseases and antimicrobial activity against foodborne pathogens [14]. All this makes these microorganisms potential probiotic candidates to reverse the toxic effects caused by LPS.

Currently, the effect of probiotic bacteria on inflammatory processes in the gut, which can be induced by the LPS, has been poorly studied. The purpose of this study was to examine the effect of probiotic supplementation with *Lactobacillus plantarum* from the gut, a mix of commercial probiotic lactic acid bacteria with five cultures of *Lactobacillus* bacteria, and also *Weissella confusa* isolated from cow’s milk on the development of LPS-induced inflammation in mice.

## 2. Materials and Methods

### 2.1. Study Objectives

Two-month-old male C57BL/6 mice were obtained from the Stolbovaya nursery (Stolbovaya, Russia) and kept at the animal facility at 25 °C and 40% humidity on a 12 h light/dark cycle. The mice were divided into five groups. The control mice (*n* = 10) group (C) received a standard laboratory diet (maintenance diet for rats and mice, Altromin Spezialfutter GmbH) throughout the entire duration of the experiment. In the 4th week, the mice received intraperitoneal saline injections for 7 days. The second group (LPS) of mice (*n* = 10) received a standard laboratory diet. In the 4th week of the experiment, the mice received intraperitoneal injections of lipopolysaccharides (LPS) (trade name Pyrogenal, N.F. Gamaleya Federal Research Center for Epidemiology & Microbiology, Russia) at 375 μg/kg/day. LPS is known to cause inflammatory responses in the gut [15]. The 3rd–5th groups of mice also received LPS injections in the 4th week of the experiment for 7 days (according to previous studies [16,17]) at a concentration of 375 μg/kg/day. The 3rd group (LA) of mice (*n* = 10) received a laboratory diet mixed with probiotic lactic acid bacteria *(Lactobacillus delbrueckii* subsp. *bulgaricus VSUET15*; *Lactiplantibacillus plantarum VSUET13*; *Lactobacillus acidophilus VSUET12*; and *Limosilactobacillus fermentum VSUET14*) from a commercial probiotic product [18,19] in a ratio of 1 × 10^8^ CFU/g of diet. The 4th (LP) and 5th (W) groups of mice received a laboratory diet mixed with *Lactobacillus plantarum VSRVR2* isolated from the gut of a 5-day-old pig (*n* = 10) and *Weissella confusa VSRVR4* isolated from cow’s milk (*n* = 10), respectively, in a ratio of 1 × 10^8^ CFU/g of diet. It was previously shown that this amount of lactic acid bacteria in the feed is considered as medium [20,21]. The average daily feed consumption by mice was 5.1 ± 0.08 g, which corresponds to each mouse consuming on average 5.1 × 10^8^ bacteria daily.

On the 29th day of the experiment, fecal samples were collected for analysis of the bacterial composition of the gut microbiome. A total of one fecal sample was collected from each mouse, which was then used for DNA extraction and sample preparation for subsequent sequencing. DNA sequencing of fecal samples using next-generation sequencing tools to assess the bacterial composition of the gut microbiome is a widely accepted practice [22,23,24]. After the course of injections, the mice were sacrificed. The mice were anesthetized with isoflurane before the sacrificing. For morphological studies of the small intestine, tissue samples were fixed in a 10–12% solution of neutral formalin HistoSafe (Biovitrum, Moscow, Russia).

The animal study protocol was approved by the Ethics Committee of FSBSI “All-Russian Veterinary Research Institute of Pathology, Pharmacology and Therapy” (protocol number 1-02/23, 10 February 2023). All procedures were in accordance with the “Animal Research: Reporting of In Vivo Experiments” (ARRIVE) guidelines and took into account the 3R principles.

### 2.2. Histological Examination

For morphological studies of the mouse small intestine, tissue samples were fixed in a 10–12% HistoSafe neutral formaldehyde solution (Biovitrum, Moscow, Russia). The intestinal tissue was dehydrated with increasing concentrations of ethyl alcohol, followed by embedding in histological paraffin “Histomix” (Biovitrum, Moscow, Russia). Sections of 3–5 µm thickness were cut from the paraffin blocks using a Hospitex Diagnostics rotary microtome. Sections were stained using classical histological methods (haematoxylin and eosin). Histological examination was performed using a Hospitex Diagnostics microscope with a built-in camera with a resolution of 5 megapixels.

Morphometric analysis was performed in the intestinal mucosa in 10 fields of view of the microscope (eyepiece 10, objective 40).

### 2.3. Whole-Genome Sequencing 

Total DNA was isolated using a commercial HiPure Microbiome DNA Kit (Magen, Guangzhou, China) according to the manufacturer’s protocol.

Sequencing was performed on the DNBSEQ-G50 platform (MGI, Shenzhen, China) according to the MGIEasy Fast FS DNA Library Prep Set protocol. The process includes fragmentation of the total DNA set MGIEasy Fast FS Library Prep Module (MGI, China), adapter ligation, and ligation of barcodes with PCR. The PCR protocol involves 95 °C for 3 min, followed by 7 cycles of 98 °C for 20 s, 60 °C for 15 s, and 72 °C for 30 s. After finishing the cycles, the samples were incubated for an additional 10 min at 72 °C. The next steps include a magnetic cleaner, monitoring the yield of the PCR product using electrophoresis in a 2% agarose gel, measuring concentration with a fluorometer, calculating the mass of the PCR product, calculating the amount of sample to be added to the pool, holding the pools, circularization of single-stranded DNA using the MGIEasy Dual Barcode Circularization Module kit (MGI, Shenzhen, China), cleaning the resulting product with magnetic particles, measuring DNA concentration with a fluorometer and QuDye ssDNA Assay Kit (Lumiprobe, Moscow, Russia), calculating the volume in μL of each pool that must be added when compiling a super pool, measurement of super pool concentration, calculating the volume in μL of the super pool covering 60 fmol, creation of DNB, measuring the concentrations of the resulting DNB using a fluorometer, and loading the cartridge.

### 2.4. Bioinformatics and Statistical Analysis

The quality of raw reads was assessed using FastQC [25]. Technical sequences and low-quality bases (Q < 30) were trimmed with fastp [26]. To remove human and mouse genome sequences, reads were mapped to reference genomes using Bowtie2 [27]. Taxonomic profiling of bacteria, viruses, and eukaryotes that form the microbiome of the studied samples was performed using MetaPhlAn4 [28]. HUMAnN3.0 was used for the functional profiling of metabolic pathways [29].

Statistical manipulations were performed in the R environment. Phyloseq was used to calculate microbiome diversity measures. The Shannon index was used to assess alpha diversity, and the Bray–Curtis dissimilarity metric was used for beta diversity. Differences in alpha diversity were evaluated using the Wilcoxon rank sum test [30]. The ADONIS function was used to estimate between-group diversity differences. Species differential abundance analysis was conducted using the MaAsLin2 package, which employed a multivariate regression model [31]. The Tukey test was used to statistically analyze the differences between groups, based on the normal distribution of the values of the morphometric parameters. A statistically significant result was considered to be an adjusted *p*-value of ≤0.05.

## 3. Results

### 3.1. Histological

**Figure 1:**
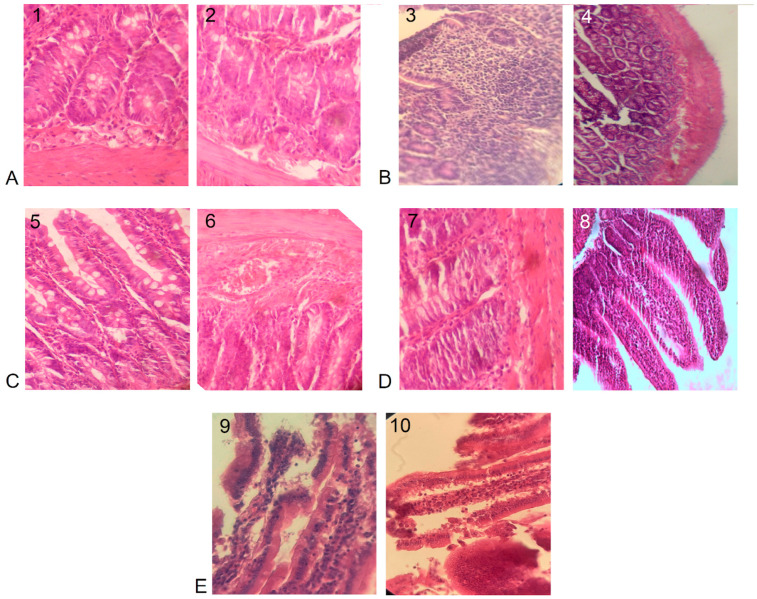
Morphological structure of a mouse intestine. Magnification: (**A**,**C**,**D**) 400×, (**B**,**E**) 100×. Hematoxylin–eosin staining: (**A**) control group, 1—villi, 2—crypts; (**B**) LPS group, 3—tissue infiltration, 4—crypts; (**C**) LP group, 5—villi, 6—vessel; (**D**) W group, 7—villi, 8—crypts; (**E**) LA group, 9—desquamation of tissue, 10—crypts.

In the control group of mice, the epithelial lining of the villi was preserved, with no signs of infiltration. The submucosa consisted of connective tissue with blood vessels. Crypts were visualized (Figure 1A).

In the LPS group, focal infiltration of lymphocytes and plasma cells with moderate neutrophil content was observed, mostly scattered in the villi. The epithelial lining of the villi was disrupted with signs of infiltration. The submucosa was represented by connective tissue with vessels in which signs of infiltration were observed. These changes were indicative of inflammation (Figure 1B).

In the LP group, the epithelial lining of the villi was preserved without signs of infiltration. The submucosa consisted of connective tissue. The crypts in the small intestine were flat and visualized. Secretion from goblet cells was observed (Figure 1C).

In group W, the intestinal architecture was the same, the mucosa had uneven oedema, the integumentary epithelium was largely preserved, in some places, the apical part of the villi was partially desquamated, and the rest of the area consisted of tall prismatic enterocytes with a well-defined brush border. The epithelial lining of the intestine was intact, and the cells were tightly packed (Figure 1D).

In the LA group, the epithelial lining of the villi was desquamated with signs of infiltration. The submucosa consisted of connective tissue. The crypts were shallow and slightly tortuous, and mitotic activity was low. Desquamation of the epithelial layer was noted in the apical part (Figure 1D).

Significant differences between the study groups were confirmed by morphometric analysis of the mice’s small intestines (Table 1).

Morphometric examination of intestinal tissue samples from the groups of mice revealed a statistically significant decrease in villus height in the LP group compared to the control group (102.27 ± 22.28 vs. 144.23 ± 23.21, respectively, *p* = 0.008). We also observed that the mean villus height in the LPS group was 121.24 ± 13.27 microns, which was lower compared to the control and some probiotic groups. At the same time, the LPS group showed a thickening of the intestinal villi of 28.72 ± 2.25 microns compared to the other study groups, and the values of the creep depth index in the LPS group were the smallest at 35.66 ± 7.65 microns. Thus, the difference between the intestinal morphometric parameters in the W, LA, and LP groups relative to the LPS group was 12.56%, and the overall difference between the LPS and control groups was 23.3%.

### 3.2. Microbiome Composition

**Figure 2:**
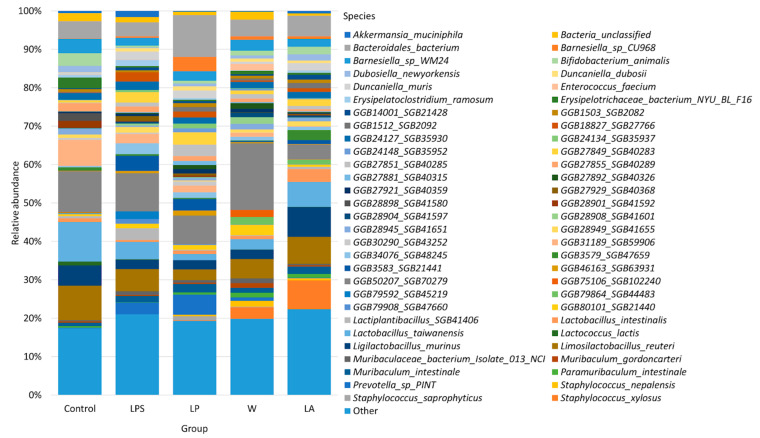
Bacterial composition of the studied samples at the species level.

Whole-genome shotgun sequencing on the DNBSEQ-G50 platform identified 446 bacterial and 2 eukaryotic species in the samples studied. In total, 290 bacterial species were identified as previously uncharacterized, reflecting the fact that the mouse fecal microbiome is a poorly studied consortium (with many uncultured and therefore few correctly named species). The abundance of 58 species was greater than 1%; they were the most abundant species in the study groups; all other species were grouped as “Others” (Figure 2).

The most abundant species in the control, LPS, and W groups was the uncharacterized *GGB50207_SGB70279* belonging to the phylum Bacteroidetes. Its abundance was 10.66% ± 0.31, 9.90% ± 0.54, and 17.34% ± 0.30 for the control, LPS, and W groups, respectively. *Lactobacillus taiwanensis* was the most commonly described species in the control group (10.30% ± 0.19), *Limosilactobacillus reuteri* in the LPS group (5.80% ± 0.01), and *Bacteroidales bacterium* in the W group (4.33% ± 0.02). The most abundant species in the LP group was also *Bacteroidales bacterium* (10.93% ± 0.16). *Ligilactobacillus murinus* was the most abundant species in the LA group (7.68% ± 0.37).

**Figure 3:**
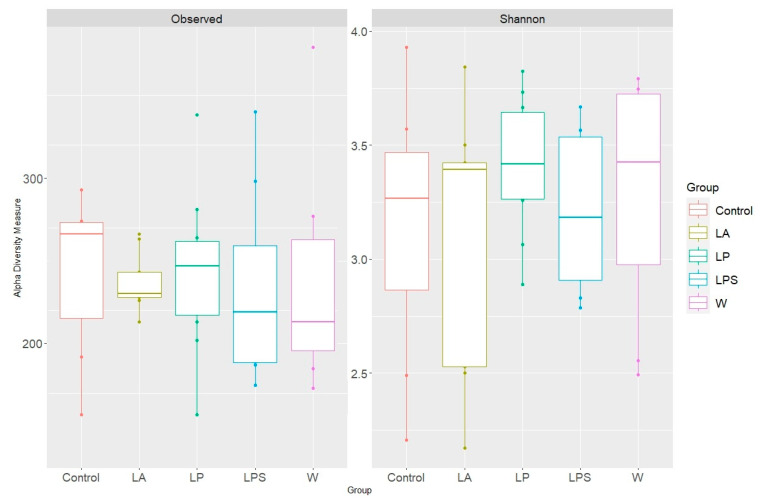
Microbiome alpha diversity of study groups.

We also conducted an alpha diversity analysis using a measure of observed species as well as the Shannon index (Figure 3).

The alpha diversity of the mouse fecal microbiome was characterized as high, as the Shannon index value was greater than three for all study groups (Table 2).

The state of the fecal microbiota was not statistically different between the groups of mice studied and can be characterized as balanced and stable due to high bacterial diversity.

**Figure 4:**
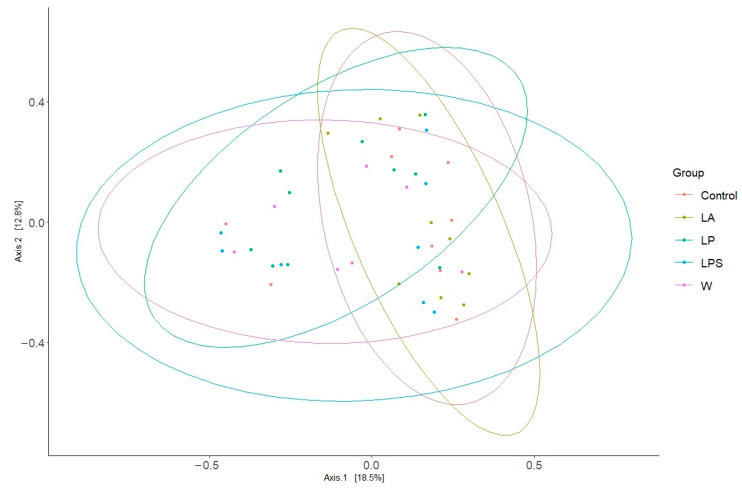
Principal coordinate analysis (PCoA) plot of the beta diversity based on the Bray–Curtis dissimilarity index.

For the study groups, we also examined the beta diversity of the mouse fecal microbiome (Figure 4).

A statistically significant bacterial clustering between the control and LP groups has been observed (*p* = 0.03).

**Figure 5:**
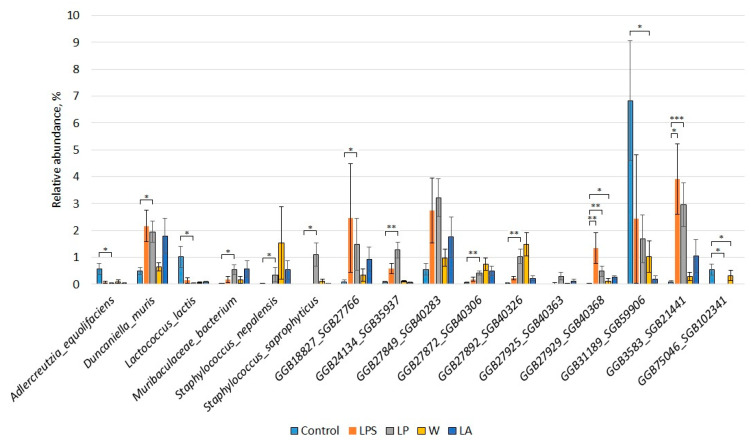
Differences in the composition of the fecal microbiome between the groups studied. * *p* < 0.05, ** *p* < 0.01, *** *p* < 0.001.

Differential abundance analysis has revealed statistically significant differences in the abundance of 16 bacterial species between the groups studied (Figure 5).

It has been found that the abundance of Adlercreutzia equolifaciens (0.04% ± 0.02 vs. 0.58% ± 0.20, *p* = 0.01), Lactococcus lactis (0.04% ± 0.02 vs. 1.02% ± 0.40, *p* = 0.02), and GGB75046_SGB102341 belonging to Eggerthellaceae (0% vs. 0.54% ± 0.20, *p* = 0.01) was lower in the LP group compared to the control group. At the same time, the abundance of Duncaniella muris (1.95% ± 0.40 vs. 0.50% ± 0.12, *p* = 0.02), Muribaculaceae bacterium (0.54% ± 0.17 vs. 0.004% ± 0.003, *p* = 0.02), Staphylococcus nepalensis (0.35% ± 0.27 vs. 0.00005% ± 0.00005, *p* = 0.03), Staphylococcus saprophyticus (1.11% ± 0.43 vs. 0%, *p* = 0.03), GGB18827_SGB27766, which is closest to Bacteroides acidifaciens (1.49% ± 0.97 vs. 0.09% ± 0.08, *p* = 0.01), GGB24134_SGB35937 belonging to Lactobacillaceae (1.27% ± 0.28 vs. 0.09% ± 0.04, *p* = 0.004), GGB27849_SGB40283 belonging to Bacteroidales (3.23% ± 0.69 vs. 0.55% ± 0.22, *p* = 0.03), GGB27872_SGB40306 belonging to Bacteroidetes (0.42% ± 0.07 vs. 0.06% ± 0.02, *p* = 0.002), GGB27892_SGB40326 belonging to Selenomonadaceae (1.04% ± 0.26 vs. 0.04% ± 0.02, *p* = 0.002), GGB27925_SGB40363 belonging to Muribaculaceae (0.30% ± 0.14 vs. 0%, *p* = 0.02), GGB27929_SGB40368 belonging to Staphylococcaceae (0.48% ± 0.20 vs. 0.0008% ± 0.0008, *p* = 0.002), and GGB3583_SGB21441 belonging to Muribaculaceae (2.96% ± 0.80 vs. 0.09% ± 0.03, *p* = 0.00009) was higher in the LP group compared to the control group. 

The abundance of GGB27929_SGB40368 belonging to Staphylococcaceae (1.35% ± 0.57 vs. 0.0008% ± 0.0008, *p* = 0.004) and GGB3583_SGB21441 belonging to Muribaculaceae (3.91% ± 1.31 vs. 0.09% ± 0.03, *p* = 0.01) was higher in the LPS group compared to the control group.

The abundance of GGB27929_SGB40368 belonging to Staphylococcaceae (0.26% ± 0.07 vs. 0.0008% ± 0.0008, *p* = 0.02) was higher in the LA group compared to the control group, but the abundance of GGB31189_SGB59906 belonging to Erysipelotrichaceae (0.18% ± 0.14 vs. 6.83% ± 2.22, *p* = 0.04) was lower in the LA group compared to the control.

Bacterial abundance in group W was not significantly different from the control.

**Figure 6:**
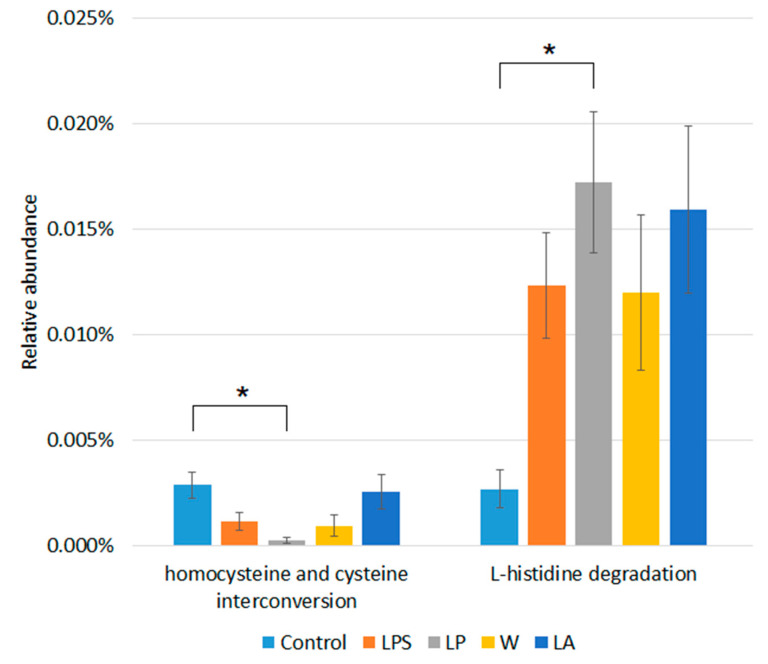
Differences in metabolic pathway abundance between study groups. * *p* < 0.05.

Profiling of metabolic pathways in the studied microbiome community was also carried out. In total, 335 functional profiles were identified. However, the frequency of unintegrated and unmapped pathways was around 97%. The statistical analysis showed that there was a significant difference in the abundance of three metabolic pathways between the control group and the LP group (Figure 6).

It can be observed that the homocysteine and cysteine interconversion pathway (0.0003% ± 0.0001 vs. 0.003% ± 0.001) was less abundant in the LP group compared to the control. On the other hand, the L-histidine degradation pathway (0.17% ± 0.002 vs. 0.003 ± 0.001) is most abundant in the LP group compared to the control (in all cases, *p* = 0.03).

## 4. Discussion

A morphological study of mouse intestinal sections showed no changes in the morphological structure of the intestine in the control group. The presence of inflammatory sites, as well as the desquamation of intestinal villi, the release of secretions from goblet cells, and hemorrhages were found in intestinal sections obtained from mice in the LPS group. A reduction in the inflammatory process in the small intestine was also seen when people took probiotic supplements containing *Lactobacillus plantarum* and a mixture of lactobacilli.

To study the mouse fecal microbiome, we used the DNBSEQ-G50 whole-genome sequencing platform (MGI, China). This approach is more sensitive to small changes, has higher resolution, and provides a more complete picture of the structure and function of host microbial communities than *16S rRNA* gene sequencing, which is used in most gut microbiota studies [32]. The microbiome composition of the study groups was characterized as rich and diverse. In addition to high numbers of bacteria, two closely related eukaryotic species were also identified in the mouse feces: *Cryptosporidium hominis* and *Cryptosporidium parvum*, although at low concentrations. *Cryptosporidium*, a ubiquitous protozoan parasite of vertebrates, causes cryptosporidiosis, a diarrheal disease that can become chronic and life-threatening in the absence of a competent immune response. *C. hominis* and *C. parvum* are known to be responsible for most human cases of cryptosporidiosis. Domestic and wild rodents have been reported to be susceptible to infection with *Cryptosporidium* spp., although they are usually asymptomatic [33,34,35]. We observed the presence of both parasite species in group W (one infected mouse). *C. hominis* was also detected in the LA group (one mouse only). No signs of infection were observed in these rodents.

Analysis of the differential abundance of bacterial species present in the fecal microbiome of mice from the studied groups showed the presence of statistically significant differences. We observed the greatest number of differences between the control and LP groups. We also observed changes in bacterial abundance between the control and LPS and LA groups. However, the bacterial composition of group W did not differ from that of the control group. We also observed no differences in the microbiome composition between the LPS group and the groups in which the mice were fed different probiotics. Thus, although there were morphological signs of intestinal wall regeneration in the LA and LP groups, we cannot isolate specific microorganisms that contributed to the pro-inflammatory and regenerative process.

The bacterium *Adlercreutzia equolifaciens*, belonging to the family *Mycobacteriaceae*, is known to contribute to the metabolism of polyphenols and is capable of forming bioactive molecules that reverse metabolic disorders in obesity and diabetes. Along with this, *A. equolifaciens* is an equol-producing bacterium and is also responsible for the production of dihydroresveratrol [36,37]. Previously, through analysis of the intestinal microbiota, it was found that, compared with a healthy control group, the number of *A. equolifaciens* bacteria decreased in a group of patients with liver diseases. This species has also been shown to decline as the disease progresses and eventually disappears in the final stages, indicating a strong association with disease severity. In addition, *A. equolifaciens* bacteria have been shown to have anti-inflammatory properties [38]. In a study of changes in the gut microbiota in men with hyperuricemia, it was shown that the bacterium *A. equolifaciens* may alleviate the damage from the disease by somehow protecting against estrogen [39]. The results of our study are consistent with previously obtained data. We showed that the bacterial species *A. equolifaciens* decreased in the group taking a bacterial supplement of *Lactobacillus plantarum* relative to the control group.

The bacterial species *Duncaniella muris* is widespread in the intestinal tract of mice [40]. It is known that this bacterium is capable of breaking down complex plant polysaccharides, such as hemicelluloses and pectins [41]. It was also previously shown that exposure to the antibiotic sulfamethazine significantly increased the relative abundance of *D. muris* bacteria in the offspring of female mice [42]. It has been previously revealed that one of the taxa that predicted the effectiveness of the combination of early inulin and pantoprazole was *D. muris*, which is known to produce short-chain fatty acids [43]. It was also noted that colonization with *D. muris* increased susceptibility to sodium dextran sulfate-induced intestinal inflammation in pathogen-free wild-type mice, a finding that may indicate that the reduction in acetate production is an indirect result of the dominance of specific Bacteroidales groups typically associated with increased inflammation after intestinal injury [44]. According to the results of our study, it was found that in the group of mice taking the *Lactobacillus plantarum* supplement, the number of the bacterial species *D. muris* was higher than in the control group. Possibly, the injections of LPS into this group of mice might be the cause.

Analysis of the results also showed an increase in the abundance of previously uncharacterized species found in the feces of mice that took a supplement consisting of *Lactobacillus plantarum*. These species were *GGB18827 SGB27766* and *GGB27872 SGB40306*, belonging to the phylum Bacteroidetes. In turn, the uncharacterized species *GGB18827 SGB27766* is closest to *Bacteroides acidifaciens*. One of the most common bacterial phyla present in the gut microbiota is Bacteroidetes [45]. Representatives of this type mostly inhabit the distal intestine, where they help provide the host with energy obtained from the diet through the fermentation of indigestible polysaccharides. This process can produce short-chain fatty acids, which provide up to 10% of daily calories if the diet is rich in fiber [46]. *B. acidifaciens* is one of the dominant commensal bacteria in the gut [47]. A study in a mouse model showed that *B. acidifaciens* had a protective effect against concanavalin A-induced liver injury [48]. Nagahara et al. (2020) showed that *B. acidifaciens* can increase intestinal immunoglobulin A levels, protect the intestine from pathogen infection, and alleviate inflammatory bowel disease [49]. Another study found that alginate oligosaccharide and its compound supplement could enhance immune function and improve intestinal microecological disorders by increasing the abundance of *B. acidifaciens* [50]. A study also showed that *B. acidifaciens* can alleviate dextran sulfate sodium (DSS)-induced colitis by reducing colonic mucosal damage, reducing inflammatory response, promoting mucosal barrier repair, and restoring intestinal microbiota diversity and balance in mice [51].

Moreover, an increase in the abundance of the uncharacterized species *GGB27849 SGB40283*, belonging to the order Bacteroidales, relative to the control group, was identified. Species of the order Bacteroidales are the most common Gram-negative bacteria in the human gut microbiota [52]. Bacteroidales have unique and active carbohydrate utilization systems and create networks of syntrophic interactions [53]. In addition, an increase in antibiotic resistance has been noted for Bacteroidales species [54].

*Lactococcus lactis* is a Gram-positive, facultative anaerobic bacterium; hundreds of strains of this bacterium are known [55]. The main beneficial effect reported is the anti-inflammatory potential of *L. lactis*, indicating its potential use as a therapeutic agent for chronic intestinal diseases [56,57]. For example, in a study by L Steidler et al., it was shown that intragastric administration of *Lactococcus Lactis*, which secretes IL-10, caused a 50% reduction in the incidence of colitis in mice treated with dextran sulfate sodium [58]. Nevertheless, this characteristic has positioned *L. lactis* as a promising probiotic and therapeutic agent. However, the pathogenicity of some *L. lactis* strains has been shown in many studies in recent years. Thus, in a study by J Antolín et al., a case of liver abscess caused by *Lactococcus Lactis cremoris* in an immunocompetent adult patient was described for the first time [59]. Some studies have shown that *L. lactis* can cause mastitis in cows, but understanding the pathogenesis of such mastitis presents a new challenge [60]. Our study showed a decrease in the number of these bacteria in the drug group compared to the control group. This may be due to the pathogenicity of some strains, but further research is needed to make a definitive statement.

*Muribaculaceae bacterium* is a strictly anaerobic bacterium that is extremely difficult to culture. It is quite widely represented in the intestines and is capable of breaking down complex carbohydrates [61]. These bacteria were found to represent a new probiotic with anti-inflammatory effects [62]. Our study showed a significant increase in these bacteria in the drug group compared to the control group, which may indicate an increase in the probiotic effect.

Our study showed an increase in the relative abundance of the uncharacterized bacterium *GGB27929 SGB40368*, belonging to the *Staphylococcaceae* family, in the LPS, LP, and LA groups compared to the control group. It is known that many representatives of this family are opportunistic microorganisms [63]. LPS can stimulate the increase in bacteria of this family, and administration of probiotics in the LP and LA groups does not appear to affect this. It is interesting to note that in group B, such a difference compared to the control group was not observed, which may indicate the influence of this probiotic on the number of *Staphylococcaceae* bacteria. However, it is difficult to say this unequivocally, since this type of bacteria, as indicated, still needs to be characterized. 

We showed that in the LP and LA groups, the uncharacterized species of bacteria *GGB75046 SGB102341*, belonging to the family *Eggerthellaceae*, was completely absent. Members of the *Eggerthellaceae* are common in the mammalian gut microbiota [64,65,66] but are rarely found in other environments. Despite having genes responsible for degrading host mucins, little is known about the role of *Eggerthellaceae* species in the gut [67]. Bacteria from the *Eggerthellaceae* family have been found to produce urolithin, have anti-inflammatory and antioxidant properties, and can also stimulate the AhR (Aryl hydrocarbon receptor) to activate tight junction proteins, thereby having anti-tumor activity [68,69]. The properties described above suggest that some members of this family could be classified as new-generation probiotics. However, we did not observe this family to become enriched in mice fed probiotic supplements.

Furthermore, the results of our experiment demonstrate that bacteria of the species *GGB31189 SGB59906*, belonging to the family *Erysipelotrichaceae*, were significantly reduced in the LA group compared to the control group. Members of the *Erysipelotrichaceae* family are widely distributed in the intestine and are associated with metabolic disorders and the development of inflammatory diseases [70]. An increase in these bacteria has been repeatedly shown in colorectal cancer [71], Crohn’s disease [72], IBD [73], and others. Thus, we can conclude that taking a mixture of lactobacilli can reduce the number of these bacteria in mice, helping to reduce the likelihood of developing inflammatory diseases, while other studied probiotics do not achieve this effect.

An increase in the abundance of *Staphylococcus nepalensis* compared to the control group was observed after the addition of *Lactobacillus plantarum* to the diet of mice. *Staphylococcus nepalensis* is a member of the genus *Staphylococcus* that was first isolated from the respiratory tract of goats with pneumonia in the Himalayan region of Nepal, and later from human urine samples, the gastrointestinal tract of squirrel monkeys, the oral cavity of domestic cats, the skin of pigs, and the environment [74,75,76]. Although *Staphylococcus nepalensis* is a normal part of mammalian flora, it needs to be studied more closely because it can cause infectious diseases and can also transfer drug-resistance genes to *Staphylococcus aureus* [74]. This study is the first to show the presence of this bacterium in the fecal microbiome of mice and its increase in response to feeding *Lactobacillus plantarum* after injection of lipopolysaccharide.

The abundance of *Staphylococcus saprophyticus* increased with *Lactobacillus plantarum* supplementation in the fecal microbiome of mice. *Staphylococcus saprophyticus* is a type of coagulase-negative staphylococci that frequently causes urinary tract infections due to its ability to produce urease [77,78]. It can also lead to the development of bacteremia, endophthalmitis, wound infections, eczema, and respiratory tract infections [79]. Peptidoglycan, isolated from staphylococci and streptococci, is also known to cause septic shock in laboratory animals. This is characterized by fever, inflammatory reactions, thrombocytopenia, and multiple-organ dysfunction syndrome [80,81]. Our study suggests the need for a more comprehensive investigation of bacterial interactions within the fecal microbiome of laboratory mice, due to an increase in the number of pathogenic representatives of the *Staphylococcus* genus when *Lactobacillus plantarum* is added to the diet.

An increase in some uncharacterized species was observed in the fecal microbiome of mice with *Lactobacillus plantarum* supplementation. The species included in the study were *GGB24134 SGB35937* from the family *Lactobacillaceae*, *GGB27892 SGB40326* from the family *Selenomonadaceae*, as well as *GGB27925 SGB40363* and *GGB3583 SGB21441* from the family *Muribaculaceae*. Also, after the injection of lipopolysaccharide, we observed an increase in the abundance of the uncharacterized species *GGB3583 SGB21441*, which belongs to the family *Muribaculaceae*. 

*Lactobacillaceae* is a family of lactic acid bacteria that inhabit the intestinal microbiota of humans and many animals. These bacteria have beneficial effects on gut health, including modulation of the immune system and protection against pathogens. Many species are used as probiotics [82]. Supplementation with *Lactobacillus plantarum* increased in a previously uncharacterized species from this family. *Selenomonadaceae* is commonly found in various anaerobic environments and is frequently isolated from the rumen and caeca of mammals [83]. This family is thought to be linked to the development of obesity [84,85]. Some studies demonstrated a negative correlation between the abundance of *Selenomonadaceae* in the human gut microbiome and *Lactobacillus plantarum* KY1032 supplementation [86]. Bacteria belonging to the *Muribaculaceae* family are known to produce propionate as an end product of fermentation and are abundant and diverse in the mouse gut. Although these bacteria are also found in humans, only a few members of this clade have been cultured and described, but their ecological role is still uncharacterized [87,88,89]. The previous study showed that *Muribaculaceae* tended to increase in abundance when a member of the *Lactobacillus* genus was added to the diet [90], which we also observed in our study for an uncharacterized member of this genus.

The study of the functional profiles of the microbiome revealed a rich diversity of metabolic pathways in the groups studied, but most of them are not integrated into databases and not mapped. This again suggests that the mouse fecal microbiome is poorly understood. Nevertheless, we observed a difference in the representation of three metabolic profiles for the LP group compared to the control group.

The trans-sulfuration pathway in bacteria is a metabolic process that converts homocysteine to cysteine through the enzymatic action of cystathionine β-synthase (CBS) and cystathionine γ-lyase (CGL) [91]. The initial step in the trans-sulfuration pathway is the condensation of homocysteine and serine catalyzed by cystathionine β-synthase (CBS). Subsequently, cystathionine γ-lyase (CGL) catalyzes the breakdown of cystathionine into cysteine, ammonia (NH3), and α-ketobutyrate [92]. The conversion of homocysteine to cysteine via the reverse trans-sulfuration pathway facilitates the conversion of methionine to cysteine, thereby providing the requisite homocysteine. Recent evidence indicates that the S-ribosylhomocysteinase (LuxS) protein, which functions as a quorum-sensing molecule in numerous bacteria, plays a pivotal role in the reverse trans-sulfuration pathway [93]. The gut microbiota can metabolize sulfur-containing amino acids, which affects homocysteine and cysteine levels. As cysteine is essential for the growth of many bacteria, the trans-sulfuration pathway facilitates the growth of microorganisms without the need for exogenous cysteine. In addition, LuxS functions as a signaling and metabolic molecule in bacteria [94]. It has been observed that dysbiosis (imbalance of the gut microbiota) in IBD patients may disrupt these pathways [94,95,96]. Some gut bacteria are capable of producing hydrogen sulfide (H_2_S) from cysteine. In moderate quantities, H_2_S has anti-inflammatory effects; however, in excess, it can be toxic and exacerbate inflammation. Additionally, numerous studies have demonstrated a close correlation between homocysteine and cysteine metabolism in various pathological conditions [97,98]. Consequently, the trans-sulfuration pathway may be implicated in metabolic dysregulation, which may in turn contribute to the development of gastrointestinal pathologies.

The L-histidine degradation pathway was predominant in the LP group compared to the control group. The ability to cleave histidine is widespread and highly conserved among all bacteria. In many cases, the complete catabolism of histidine to glutamate, ammonia, and formate or formamide allows the use of histidine as a carbon or nitrogen source or both. In some cases, cells use intermediates and pathway components to generate important signaling molecules that influence properties such as virulence or biofilm formation. The strong conservation of the enzymes involved and the genetic clustering of histidine utilization pathway genes suggest horizontal transfer over evolutionary time [99,100]. The major contribution to the representation of this pathway was made by unclassified bacteria, but it was also found in the bacteria *Parabacteroides distasonis*, *Staphylococcus nepalensis*, *Staphylococcus saprophyticus*, and *Staphylococcus xylosus* in the LP group. *Parabacteroides distasonis* is a member of the intestinal microbiota that has recently received much attention due to the many beneficial properties it confers on its host, manifesting anti-inflammatory properties and activity in reducing body weight gain, hyperglycaemia, and hepatic steatosis [101].

## 5. Conclusions

The objective of this study was to investigate the changes that occur in the gut microbiome of mice following injections of LPS, which cause inflammation. At the same time, we observed changes in the microbiota, characterized by a significant increase in the abundance of uncharacterized species belonging to *Staphylococcaceae* and *Muribaculaceae*, caused by LPS injections.

We also investigated the potential anti-inflammatory effects of some probiotic bacteria as a dietary supplement against LPS injection. No significant changes were observed in the microbiome when probiotics were administered compared to the LPS group that did not receive probiotic correction. However, at the morphological level, positive changes were observed in the gut in the presence of probiotics. *Lactobacillus plantarum* demonstrated the greatest protective effect and microbiome changes. Additionally, the greatest impact of this probiotic species was indicated by a change in the enrichment of metabolic pathways. A decrease in the enrichment of the homocysteine and cysteine interconversion pathway was observed, accompanied by a simultaneous increase in the L-histidine degradation pathway. In addition, an anti-inflammatory effect was observed in the LA group of mice, which had been administered a commercial probiotic mix of probiotic lactic acid bacteria. This effect was observed at the morphological level, but it was less pronounced than that observed in mice administered with *Lactobacillus plantarum*. In addition, histological analysis revealed that the *Weissella confusa* group did not exhibit anti-inflammatory activity, and no significant microbiome alterations were observed.

This study identified microbiome imbalances associated with LPS-induced inflammation and explored how specific probiotic bacteria can improve gut mucosal healing.

## Figures and Tables

**Table 1:** Mouse small intestine morphometric study.

Metrics, Microns	Control	LPS	LP	W	LA
Villi height	144.23 ± 23.21	121.24 ± 13.27	102.27 ± 22.28 **	143.24 ± 10.46	131.37 ± 12.89
Villus thickness	12.34 ± 2.28	28.72 ± 2.25	12.37 ± 1.23	14.33 ± 0.25	18.24 ± 1.47
Crypt depth	62.89 ± 12.59	35.66 ± 7.65	57.81 ± 4.56	58.43 ± 3.77	55.67 ± 10.26
Crypt width	17.64 ± 0.55	14.67 ± 1.21	18.25 ± 4.75	17.01 ± 2.87	25.49 ± 2.64
Brush border height	0.87 ± 0,92	0.47 ± 0.44	0,85 ± 3,77	0.82 ± 0.32	0.52 ± 0.26

** *p* < 0.01. Statistically significant differences were found only compared to the control group.

**Table 2:** Fecal bacteria numbers and their diversity measures.

Feed Treatments	Observed Species	Shannon Index
Control	276	3.15
LPS	295	3.21
LP	301	3.41
W	327	3.28
LA	269	3.12

## Data Availability

Metagenome data are available in the NCBI BioProject database (BioProject ID: PRJNA1109598).
